# The impact of uninformative parafoveal masks on L1 and late L2 speakers

**DOI:** 10.16910/jemr.13.6.3

**Published:** 2020-08-26

**Authors:** Leigh B. Fernandez, Christoph Scheepers, Shanley E.M. Allen

**Affiliations:** Technische Universität Kaiserslautern, Germany; University of Glasgow, UK

**Keywords:** eye-movement, eye tracking, parafoveal processing, individual differences, N+1 effect, bilingualism, sub-lexical orthographic information, cross-linguistic influence

## Abstract

Much reading research has found that informative parafoveal masks lead to a reading benefit for native speakers (see 1). However, little reading research has tested the impact of uninformative parafoveal masks during reading. Additionally, parafoveal processing research is primarily restricted to native speakers. In the current study we manipulated the type of uninformative preview using a gaze contingent boundary paradigm with a group of L1 English speakers and a group of late L2 English speakers (L1 German). We were interested in how different types of uninformative masks impact on parafoveal processing, whether L1 and L2 speakers are similarly impacted, and whether they are sensitive to parafoveally viewed language-specific sub-lexical orthographic information. We manipulated six types of uninformative masks to test these objectives: an Identical, English pseudo-word, German pseudo-word, illegal string of letters, series of X’s, and a blank mask. We found that X masks affect reading the most with slight graded differences across the other masks, L1 and L2 speakers are impacted similarly, and neither group is sensitive to sub-lexical orthographic information. Overall these data show that not all previews are equal, and research should be aware of the way uninformative masks affect reading behavior. Additionally, we hope that future research starts to approach models of eye-movement behavior during reading from not only a monolingual but also from a multilingual perspective.

## Introduction

During reading we take in information not only from the word we are directly fixating on, but also from words that we are not directly fixating on. Our central visual field, where visual acuity is the highest, extends approximately 2 degrees of visual angle from a fixation (i.e., the foveal area). However, we are also able to extract information extending up to 5 degrees of visual angle from the fixation (i.e., the parafoveal area; e.g., 2). Processing of information in the parafoveal area has been traditionally tested using the gaze contingent boundary paradigm (GCB; 3) in which a word of interest is first masked and the word is changed to the critical word only once the reader makes a saccade across an invisible boundary placed before the critical word. In a sentence like A.1 the critical word *ed* is made parafoveally unavailable by a mask (e.g., *xxxxxx*), and the invisible boundary is embedded between *o* and *x* in fox (marked with an *). When the reader’s gaze crosses this boundary, the non-word is permanently replaced with the critical word *jumped* as shown in A.2.

A.1 The quick brown fo*x xxxxxx over the lazy dog.

A.2 The quick brown fo*x jumped over the lazy dog.

The change that occurs from mask to critical word is triggered by a saccade, and is typically undetected by the reader due to saccadic suppression (4). The GCB allows us to test whether the reading time of *jumped* differs depending on whether it was masked (preventing parafoveal processing) as in A.1 or not (allowing parafoveal processing) as in A.2. Research has found that reading times are shorter when the word is available parafoveally [A.2] compared to when it is not [A.1]; this has traditionally been called a “preview benefit” (e.g., 1). This preview benefit suggests that readers preprocess parafoveal information while fixating on a foveal word, and this in turn facilitates reading when fixating on the next word.

The types of parafoveal masks used in the GCB paradigm can be uninformative or invalid (i.e., share no features with the critical word, like the x’s in A.1) or can be informative or *valid* (i.e., share some features of the critical word, like in B below). Other types of uninformative mask types include an illegal letter string A.3 a psuedoword A.4 or an unrelated word A.5.

A.3 The quick brown fo*x pvnqwm over the lazy dog. (illegal string)

A.4 The quick brown fo*x nuncer over the lazy dog. (pseudo-word)

A.5 The quick brown fo*x public over the lazy dog. (unrelated word)

Informative parafoveal masks share features with the critical word. In B, for example, the first three letters are shared between the mask and the critical word (*jumped*); this is called an orthographic mask.

B The quick brown fo*x jumyrb over the lazy dog. (orthographic)

It has been found that readers can preprocess orthographic, phonological, lexical, and in certain circumstances and languages even morphological and semantic information parafoveally (for a review see 1). For example, when a parafoveal mask shares the first few letters of the critical word as in B, there is a large preview benefit. Balota, Pollatsek, and Rayner (1985) found that having the parafoveal masks cake or cahc (for the critical word cake) yielded larger reading time preview benefits compared to when the mask was pies, picz, or bomb (5). This effect was particularly pronounced when the target word was highly predictable based on the context of the sentence. In a recent meta-analysis including 88 studies that have investigated preview benefits, Vesilev and Angele (2017) found that parafoveal masks that contained useful orthographic, phonological, or semantic information facilitated reading times for the critical word (i.e., preview benefit), with orthographic masks leading to the greatest facilitation (and semantic the least facilitation) compared to masks with no useful information (6).

Uninformative masks, on the other hand, lead to a “preview interference” effect such that the less “word-like” the mask, the more the interference. For example, Hutzler et al. (2013) had participants read a list of five words (written in a line) and make a judgment about whether the last word in the list was given previously in the list or not (7). In the masked conditions they blocked the last word with a series of X’s and embedded an invisible boundary after the second to last word (i.e., blocking the last word from the parafovea). They found a delayed emergence of an old/new effect in brain activity at the foveal word when the parafoveal word was masked compared to when the ­words were presented without a preview and no parafoveal processing could occur (words were presented individually). Marx, Hawelka, Schuster, and Hutzler (2015) found that X and illegal letter string parafoveal masks led to a larger preview interference compared to a baseline mask that was less visually salient with children, and they argued that X and illegal letter mask lead to an overestimate of the preview benefit (8)­. Furthermore, Hutzler, Schuster, Marx and Hawelka (2019) found evidence, in a series of 4 experiments with adults, that parafoveal masks can have a hidden preview cost that leads to an overestimated preview cost both in single words and in sentence reading (9). Vasilev and Angele (2017) point out that this interference seems to amount to several milliseconds for first-pass measures, but they also point out that this estimate is an exploratory finding and they encourage systematic investigation (6). Given the evidence that parafoveal masks can lead to either a preview benefit or interference Vasilev and Angele use a more neutral term - N+1 preview effect (with N referring to the word being fixated on and the +1 referring to word in the parafovea) - to talk about parafoveal processing effects (6).

Research has clearly shown the importance of parafoveal processing for native speakers, and that denying native speakers parafoveal information can negatively impact on their reading behavior. The type of mask used is particularly important to consider when calculating N+1 preview effects, because any processing costs associated with an uninformative mask may inflate reading times and potentially obscure N+1preview effects. For example, if the reading time on *jumped* when masked with a string of X’s [A.1] is 450ms, but reading times on *jumped* when masked with a string of letters [A.3] is 400ms, it may be that the masks, while both uninformative, are inflating N+1 effects differently. Additionally, despite its importance for native speakers, parafoveal processing has been largely ignored in the second language reading literature. In order to understand the process of reading in a second language, we must understand whether non-native speakers are capable of using parafoveal information, and if so, what sort of information can they extract parafoveally.

The remainder of the introduction will be divided into four sections: the first three will correspond with our three research objectives, and the fourth will introduce the study. Our first objective is to systematically test the role of different types of uninformative parafoveal masks on N+1 reading behavior. Therefore, Section 1 explores L1 parafoveal processing research focusing primarily on research with uninformative masks. Our second objective is to test whether uninformative parafoveal masks have the same impact on native speakers of English (L1) as they do on late second language English speakers (L2) with a L1 of German. Therefore, Section 2 explores L2/bilingual parafoveal processing research. Given the dearth of L2 parafoveal research, it will include all available research. Our third objective is to test whether readers are sensitive to language-specific sub-lexical orthographic information in the parafovea, to test whether L1 and L2 speakers are sensitive to native language specific information parafoveally. Therefore, Section 3 explores the potential role of language-specific sub-lexical orthographic information on parafoveal processing. Additionally, in section 3 we briefly discuss individual differences that may impact efficient extraction of parafoveal information.

### L1 Parafoveal Processing

As discussed earlier, native speakers are able to make use of orthographic, phonological, and potentially morphological semantic information parafoveally, which leads to a preview benefit or an N+1 facilitation effect. In addition, when the parafoveal mask is uninformative, this leads to a preview cost or an N+1 inhibition effect, with inhibition increasing the less “word-like” the mask becomes. Indeed, Kliegl et al. (2013) found that fixations on N+1 following an uninformative mask (a random string a letters) were greater the closer the prior fixation was to N+1 since the greater proximity allowed for more parafoveal processing to occur, and thus more interference from the uninformative mask (10). This led the authors to argue that the N+1 effect is a combination of preview benefits and costs. Given that the objective of the current study is to test uninformative masks, this section will focus primarily on research that has reported a comparison between different types of uninformative masks on parafoveal word. This will highlight the potential differential impact that properties of uninformative masks can have on processing (for a more comprehensive overview of L1 parafoveal processing see 1; We will not discuss further the role of visual salience of parafoveal masks (see for example; 9 or 10).

Using the GCB paradigm, Slattery et al. (2011) had participants read sentences with different types of parafoveal masks and judge whether they saw something change while reading the sentence (display change detection paradigm) (11). Participants read sentences with differing case (upper vs. lower), such as BoYs’ voices WiLl noticeabl*y ChAnGe during PuBeRtY. Across two studies, the parafoveal mask of the critical word ChAnGe was varied by case and/or letter (ChAnGe/cHaNgE), string of letters (RbEcPa/rBeCpA), real (but unrelated) word (AlWaYs/aLwAyS), and non-word (ElWaYs/eLwAyS). In addition, the time in which in the parafoveal mask changed to the critical word occurred either immediately (~8ms) after making the saccade across the invisible boundary (indicated with *) or after a delay (15 or 25 ms). Slattery et al. found an interaction that is particularly relevant for the current study: in the delayed condition, when the parafoveal mask contained a different case than the critical word there was a greater effect of real word masks (AlWaYs/aLwAyS) relative to non-word masks (ElWaYs/eLwAyS). This suggests that properties of uninformative parafoveal masks can directly influence fixation durations on the critical word.

In a study looking at the impact of the parafoveal word on foveal processing, Angele, Tran, and Rayner (2013) manipulated the parafoveal word (once) in sentences like Victor read the news* once this morning to test whether reading of the foveal word (news) was affected (12). While the main aim of their paper was to investigate how the foveal word (news) was impacted by the parafoveal word (once), pertinent for the current study, eye-tracking measures on the parafoveal word (once) were also reported. In their first study they had the identical parafoveal word (once), a repetition of the critical word (news), an unrelated preview (warm), and a non-word preview (rzmc). In the second study they again had the identical parafoveal word (once), a repetition of the critical word (news), but additionally had an orthographically related preview (niws), a semantically related preview (tale), and a non-word preview (tule). In both of their experiments the identical preview served as the baseline with which they compared all other masks.

While the authors did not make any additional comparisons (apart from the masked conditions being individually compared to the baseline), inspection of the reported linear mixed models for the parafoveal word in Experiment 1 suggested that fixations, probabilities of being fixated upon, and likelihood of a regression out of the parafoveal word (once) were greater when masked by a non-word (rzmc) relative to an unrelated word (warm). In Experiment 2 the durations on the critical word when masked with the non-word mask (tule) were slightly shorter across early reading measures relative to the orthographic mask (niws). While there were no inferential statistics confirming these patterns, the findings again highlight that properties of parafoveal masks may influence the processing of the critical word. The authors discussed the possibility that the unusual letter string in niws disrupts processing more than the letter string in tule, which is more like a pronounceable pseudo-word.

Another study using the GCB paradigm combined with display change detection (13) manipulated the word-likeness of the masks, in sentences like She designed the peaceful* garden behind her house herself. The authors manipulated the parafoveal masks on the critical word garden, using a word-like mask (puvtur) or a non-word-like mask (xbtchp). They found that the type of uninformative mask did not lead to a significant N+1 preview effect on the target word; gaze duration (the duration of fixations on the critical word before the eye moves to another word) on the critical word (garden) when the parafoveal word was puvtur was 342ms, compared to 354 ms when the word was xbtchp. However, they did find that participants were more sensitive to noticing the change from a non-word-like mask to the critical word relative to when the change was from a word-like mask to the critical word. Results from (12) and (13) led Angele and colleagues to suggest that parafoveal processing is two-staged. In the first “early” stage, readers may engage in a visual check in which reading is monitored; this is a preattentional orthographic stage. In the “late” second stage, readers may engage in deeper attention-dependent processing in which lexical processing occurs.

While the N+1 preview effect has been well researched, and there is evidence of an inhibitory effect of uninformative relative to informative parafoveal masks, it still remains unclear as to how different types of uninformative masks may influence processing. In their meta-analysis, Vasilev and Angele argue for a graded effect, with reading interference increasing the less word-like a mask becomes (6). That is, interference increases as follows: unrelated word < pseudo word < random string of letters < string of Xs. While this is not in line with the lack of interference differences between pseudo-words and a string of letters (puvtur vs. xbtchp; 13), it is in line with the potential interference from a string of letters relative to a pseudo word (niws vs. tule; 12), and unrelated real-word relative to the non-word (AlWaYs/aLwAyS vs. ElWaYs/eLwAyS; 11). For the latter two studies, the pattern of results lent itself well to the graded effects reported by Vasilev and Angele (6). It is important to note that the previous studies were not designed to investigate different types of uninformative masks (and in some case are not statistically tested), and N+1 interference may only amount to a few milliseconds (6). Therefore, in the current study we aim to systematically test how the properties of uninformative masks influence processing.

### L2/Bilingual Parafoveal Processing

Research investigating bilingual and L2 speakers’ parafoveal processing using the GCB has primarily focused on the semantic level. Several studies have investigated the amount of parafoveal information necessary for L2 speakers to read typically, but these studies did not directly manipulate individual word level aspects using the GCB paradigm and will therefore not be discussed further (see 14, 15, 16, 17, 18, 19). To the knowledge of the authors, there is no research using, specifically, non-word parafoveal masks with L2 speakers (while the research outlined here does make use of uninformative parafoveal masks, they were always real words within the language, and no other types of uninformative masks were used).

Altarriba, Kambe, Pollatsek, and Rayner (2001) compared parafoveal processing in Spanish-English bilinguals while reading in both English and Spanish (20). They found that bilingual participants showed no preview benefit as a result of a semantic mask when presented with a parafoveal mask that was a direct, but non-cognate translation of the critical word (that is, the critical word and mask had the same meaning but did not overlap in orthographic or phonological features, as in fuerte (strong) as a parafoveal mask of strong during reading of English). However, they did find a preview benefit when the mask was a cognate of the critical word (such that both had the same meaning and shared orthographic/phonological features), as in crema (cream) as a parafoveal mask of cream. A preview benefit even emerged when the mask was a ‘psuedocognate’ of the critical word (such that both had different meanings but shared orthographic/phonological features) as in grasa (grease) as a parafoveal mask for grass. This suggests that the preview benefit was orthographic/phonological in nature rather than semantic, as there was no benefit in the non-cognate semantic overlap condition (fuerte/strong). Therefore Spanish/English bilingual speakers seem to derive an N+1 facilitation from orthographic/phonological parafoveal information, but derive no such N+1 facilitation from semantic information presented parafoveally.

More recently, Wang and colleagues tested parafoveal processing by L1 Korean speakers reading in their L2 Chinese (21, 22). In one study Wang et al. (2016) tested L1 Korean speakers, who had been studying Chinese for an average of 3.9 years and were undergraduate students in Beijing (21). They read sentences in Chinese with Korean parafoveal masks. The masks were either a cognate translation preview (identical meaning and similar pronunciation, but without orthographic overlap), a related preview (semantically related but not phonologically related), or an unrelated preview of the Chinese critical word. They found an N+1 cognate facilitation effect as well as an N+1 semantic (related preview) facilitation effect; given that the latter had no orthographic or phonological overlap, it can be interpreted as a pure semantic benefit.

The lack of a semantic N+1 facilitation effect seen by Spanish/English bilinguals (relative to the semantic N+1 facilitation effect seen for Korean/Chinese bilinguals) may stem from the fact that semantic information is available relatively late in English due to the opaque orthography, while in logographic languages, like Chinese, sound and meaning are more closely mapped orthographically, leading to more direct access to semantic information (21). Wang et al. note that, similar to the Altarriba et al. study discussed earlier, their goal was to test semantic parafoveal processing rather than bilingualism (20).

Wang et al. (2014) tested L2 parafoveal processing more directly by investigating the role of L2 reading proficiency on L2 parafoveal processing in L1 Korean/L2 Chinese speakers (22). Participants read a series of two-character words as quickly as possible, and the average number of correctly named words per minute served as a measure of reading proficiency. They then read Chinese sentences in which a critical word was masked with either an identical mask, an orthographically related mask, a phonologically related mask, a semantically related mask, or an unrelated mask (masks were in Chinese). They found that the L2 speakers only showed N+1 facilitation effects when the mask was identical or orthographically similar to the critical word, and that this facilitation was greater for those participants with a higher reading proficiency score. The authors argue that L2 speakers may only be capable of extracting visual information from the parafovea given that there was no N+1 facilitation at the phonological or semantic level. In conjunction with their previously discussed study (21), the authors speculate that higher level parafoveal processing is most likely influenced by factors at the visual level, linguistic level, and individual level.

Taken together, these studies suggest that L2 speakers are capable of making use of parafoveally presented orthographic information regardless of the language (20, 21, 22), and that this facilitation is modulated by L2 proficiency (22). Higher level semantic parafoveal processing was only seen in L2 speakers of a logographic language when the mask was presented in their L1 (21). Given that the objective of the current study is not to test meaningful parafoveal masks, but rather uninformative parafoveal masks, we will not discuss this further. What these studies highlight, however, is that L2 speakers are able to use parafoveal information, and that the three groups in the studies outlined above all showed (at the minimum) the ability to extract visual level features from the parafovea that are relevant for L2 processing. This seems to correspond to the pre-attentional orthographic stage as suggested by Angele and colleagues (12, 13). Additionally, the Wang et al. (2014) study highlights the role of reading proficiency in the ability to use parafoveal information (22).

### Sub-Lexical Orthographic Information & Individual Differences

In addition to testing the role of uninformative parafoveal masks and whether L1 and L2 processing are similarly affected by uninformative masks, we are also interested in whether readers are sensitive to parafoveally presented sub-lexical information that is specific to their native language (i.e., are German speakers more sensitive to “German-like” pseudo-words than they are to “English-like” pseudo-words?). As discussed previously, descriptive statistics in Experiment 2 of Angele et al. (2013) revealed that parafoveally viewed words that were masked with a non-word (tule) had slightly shorter durations than when masked with an orthographic mask (niws) (12). The authors suggest that the pronounceable non-word may show less of an inhibitory effect than the orthographic mask, which could be treated as some sort of illegal/unusual string of letters. Given that this pairwise analysis was not made (nor was it the aim of their study) and the difference between reading durations after each mask type was relatively small, we do not know whether readers are sensitive to parafoveal language-specific sub-lexical orthographic information. It has also been found that L1 readers of Chinese were able to make use of sub-lexical semantic information that was viewed parafoveally, suggesting that sub-lexical information can be extracted parafoveally at least in languages like Chinese where sound and meaning are more closely mapped orthographically (23). Thus, we aim to test this directly in the present study.

Research has shown that individual differences, particularly in proficiency and in the quality of lexical representation (i.e., knowing a word’s orthographic, phonological, semantic, and syntactic qualities; 24), can impact on the efficiency with which L1 and L2 speakers can extract parafoveal information. For example, Veldre & Andrews (2014) found that L1 speakers with higher quality of lexical representation (as measured by spelling skills) and higher reading skills (as measured by a vocabulary and reading comprehension test), showed a greater benefit from the availability of valid parafoveal information relative to lower scorers (25). In addition, they found that skilled readers were more negatively affected by uninformative parafoveal information; that is, reading durations and saccade length decreased when parafoveal information was denied. Therefore, they argue that the higher the quality of lexical representation, the more efficiently readers are able to identify words in the parafovea, extract information, and program upcoming eye movements. Whitford & Titone (2015) also found that higher quality of lexical representation (as measured by L2 exposure) in a second language facilitates parafoveal processing (19).

As discussed in section 2, Wang et al. (2014) found direct evidence that L2 speakers with higher proficiency were more efficient at extracting parafoveal information (22). While Whitford and Titone found an impact of L2 exposure on the ability to extract parafoveal information, they did not include proficiency in their analysis. It is not difficult to assume that more exposure to an L2 would also lead to higher proficiency, and in the same light better spelling skills, both of which would likely impact the ability to extract parafoveal information (25). Therefore, in the current study we control for both of these potentially important sources of individual differences by adding a measure of proficiency (based on morphosyntax) and a measure of quality of lexical representation (based on spelling skill) as predictors in our statistical models.

### The Present Study

The studies outlined above show that parafoveal masks can affect the subsequent reading times on word N+1, with informative masks leading to a facilitation effect, and uninformative masks leading to an inhibition effect. However, it remains unclear how different properties of uninformative parafoveal masks affect reading behavior. In addition, research investigating parafoveal processing has been primarily restricted to native speakers, with very little research investigating parafoveal processing in bilingual and/or second language speakers. Therefore, in the current study we test several types of uninformative masks with two groups of speakers, monolingual L1 speakers of English, and late L2 speakers of English (with an L1 of German). This allows us to test how different properties of uninformative parafoveal masks impact on the early pre-attentional “visual check” stage (12, 13) and to directly test whether there is a graded inhibition on the N+1 word as the mask becomes less word-like. In addition, we are contributing to the limited research on parafoveal processing by L2 speakers.

Based on their meta-analysis, Vasilev and Angele (2017) suggested the following graded interference from uninformative parafoveal masks: unrelated word < pseudo-words < random strings of letters < string of X’s (6). In the current study, the degree to which uninformative masks interfere with reading times on N+1 was investigated using five uninformative mask types: (b) an ‘English-like’ pseudo-word mask, (c) a ‘German-like’ pseudo-word mask, (d) a string of random letters, (e) a row of X’s, and (f) a mask with no visual information (blank space). The two pseudo-word masks in particular allow us to test whether readers are sensitive to language-specific sub-lexical information in the parafovea. It may be that the L1 and L2 speakers treat all pseudo-words the same regardless of the language they are derived from (showing similar interference effects for both pseudo-word types), or it is possible that they are more sensitive to pseudo-words derived from their own language. The blank mask was used to test whether having pure whitespace in the parafovea impacts N+1 reading times. It is possible that the blank mask will lead to small interference effects given that no orthographic information will have been available to process, or it is possible that the blank mask will lead to large interference effects given that readers will be aware of the change within the sentence and they have no information about the word. We tentatively hypothesize a greater interference from the blank mask. Therefore, we hypothesize the least interference with pseudo-words and the most interference with strings of Xs or Blank. The predicted pattern therefore looks like: Pseudo-words (potentially dependent on L1 compatibility) < Illegal Strings < String of Xs ≤ Blank (where ‘<’ is taken to indicate less interference).

In the current study we test language-specific sub-lexical orthographic information by using pseudo-words from Schröter and Schroeder (2018) as parafoveal masks (26). They created a set of more “German-like” pseudo-words and a set of more “English-like” pseudo-words. While Adults have been shown to be sensitive to this type of manipulation, Schröter and Schroeder found that bilingual children were not sensitive to this language-specific sub-lexical manipulation in a seemingly monolingual lexical decision task (i.e., when deciding whether a string of letters is a word or not in German there was no difference in decision speed as a result of the language in which the psuedoword was derived) (26). In terms of adults, Lemhöfer and Radach (2009) tested adult unbalanced bilinguals (L1 German, L2 English) and found slower decisions to more, what they called English-like non-words (but were similar to the pseudo-words from 26.), than to what they called German-like non-words in a mixed-language lexical decision task (27). The authors argued that when a stimulus forms a non-word in the weaker language (L2) of a bilingual, it takes longer to recognize it as such compared to a non-word in the stronger language. Therefore, in the current study two types of pseudo-word masks, based on the sub-lexical orthographic information of either English or German, were used to test whether L1 and L2 speakers of English are sensitive to the language-specific sub-lexical information in the parafovea.

## Methods

### Participants

#### L1 English

Fifty-five native speakers of English were recruited from the University of Glasgow. Of those, two participants were excluded due to early exposure to a second language, and an additional two participants were excluded because they had experience with German at school (we specifically focused on participants who had no formal experience with German, since this may influence sensitivity to the German pseudo-word masks). The remaining 51 participants had spoken English from birth, had not learned a second language before the age of 5, and had not learned any German. All had normal or corrected-to-normal vision and no participant reported a language related disorder. Participants were paid 10 GBP for their participation. See Table 1 for additional participant information.

**Table 1.** Participant Information

N	L1	Male/ Female	Mean Age	Mean OPT (in English)	Mean Spelling Score (in English)
51	English	14/37	23.45 (4.12)	95.82 (4.34)	83.18 (8.13)
51	German	32/19	24.97 (3.36)	79.44 (10.07)	77.11 (8.23)

#### L2 English (L1 German)

Fifty-one native speakers of German, who were late second language learners of English were recruited from Technische Universität Kaiserslautern, Kaiserslautern, Germany. Mean age of English acquisition was 10.2 years of age (sd = 1.6; range = 6-15 years), and no participant had exposure to a second language before the age of 6. All had normal or corrected-to-normal vision and no participant reported a language related disorder. Participants were paid 10 Euro or given course credit for their participation. See Table 1 for additional participant information.

### Materials

All sentence materials were in English. The experiment consisted of 104 trials: 4 practice trials, 24 critical items, and 76 filler trials (24 of the fillers were critical items for a different study not presented here). The critical items were always shown in the second half of the study. An example of the critical stimuli can be seen in Table 2, see Appendix 1 for all critical items.

The critical stimuli started with an article The, a noun that denoted an occupation (geologist), followed by a verb (found), followed by an additional article the (where the invisible boundary was embedded, (*) in Table 2), then the critical word (rock) or one of the parafoveal masks, and a spillover region (in the cave).

**Table 2.** Example stimuli

	Identical	English pseudo-word (EPW)	German pseudo-word (GPW)	Illegal	X	Blank
The geologist found th*e	rock	mish	mand	nhpl	xxxx	

The critical words (Identical masks) were controlled for in length across items ranging from 4 to 7 characters, for syllable count across items (critical words were either one or two syllables), stress (the same syllable was stressed), and all were high frequency words (i.e., occurred more than 20 times per million words according to the Corpus of Contemporary American English). Additionally, the critical word was expected based on the context of the sentence. The expectation of the items was established using an offline questionnaire rated by 81 native speakers of English who did not participate in the main study. Raters were asked to indicate how expected an underlined word was given the context of the rest of the sentence on a scale of 1 (least expected) to 7 (most expected). The items in the survey were the first NP, verb, and critical NP in Table 2 (the spillover region was not included). Four of the 24 original items were not rated as expected or unexpected and were removed, and an additional 4 items were created and were rated by another set of 20 native speakers of English (who did not participate in the current study) in an offline questionnaire (in the same format). The twenty items from the first survey had a mean rating of 5.95 (sd=1.39), and the four items from the second survey had a mean rating of 6.17 (sd=1.01), indicating that all items were expected.

As mentioned above there were 5 types of uninformative masks: English pseudo-word (EPW), German pseudo-word (GPW), random string of letters (Illegal), a row of X’s (X), and a blank mask (Blank). All masks were matched in length to the identical word. The pseudo-word masks were taken from Schröter and Schroeder (2018) (26); as reported previously, the authors used Wuggy (28) to generate pseudo-words from English and German nouns that were matched in length and frequency. Schröter and Schroeder separately produced pseudo-words for each language and they verified the pseudo-words using two measures of orthographic neighborhood, such that each pseudo-word was lexically similar to the language it was based on (26). In the current study, the German and English pseudo-word began with the same initial letter within each item.

The materials were constructed according to a one-way design with six levels, such that the critical word was preceded by one of six different mask types (Identical, EPW, GPW, Illegal, X, or Blank). Overall, the study was a 2x6 design, crossing language (English, German) and mask type (6 levels). Language was between-subjects but within-items, and mask type was both within-subjects and within-items. All sentences were presented on a single line in Courier New font (monospaced) with a font size of 20.

### Apparatus

#### L1 English

Stimulus presentation was programmed using Experiment Builder, and eye movements were recorded using an Eyelink 1000 sampling at 1000 Hz. Viewing was binocular, but only the right eye was recorded. The head was stabilized using a chin rest, and participants sat approximately 72 cm from the screen. Stimuli were presented on a Dell P1130 19” flat screen cathode ray tube (1024 X 768 resolution; 150 Hz refresh rate) and approximately 2 characters subtended 1° of visual angle. The refresh rate yielded a mean display change of 6.21 msec (sd – 2.12 msec).

#### L2 English

Stimulus presentation was programmed using Experiment Builder, and eye movements were recorded using an Eyelink 1000 or EyeLink Duo sampling at 1000 Hz (note: The Eyelink 1000 was replaced by an Eyelink Duo due to a malfunction in the host computer. The program, setup, display computer, display screen, and room remained the same across the entirety of the study. A total of 31 participants were run using the Eyelink1000, and 20 with the Eyelink Duo. There were no notable differences across the two machines in terms of average first fixation duration, gaze duration or skipping rate (all ps > .4). Viewing was binocular, but only the right eye was recorded. The head was stabilized using a chin rest. Due to the lab configuration, participants sat slightly further away from the screen than the L1 participants - approximately 90 cm. Stimuli were presented on a Samsung SyncMaster 959NF 19” flat screen cathode ray tube (1024 X 768 resolution; 120 Hz refresh rate) and approximately 2.4 characters subtended 1° of visual angle. The refresh rate yielded a mean display change of 6.14 msec (sd – 2.47 msec) across both machines (the Eyelink Duo had a mean display change 6.04 ms, and the Eyelink 1000 had a mean display change of 6.20 msec).

### Procedure

Procedure was identical for both language groups. Participants first went through a series of paper tasks: a language background questionnaire, the Oxford Placement Test (OPT, Part A) to assess English proficiency, and a misspelling identification task. Then they took part in the eye tracking task. Altogether, the session took approximately 60 minutes. See Table 1 for scores on the latter two tasks.

Participants were calibrated on a 9-point-calibration screen for the eye tracking tasks, and were instructed to read silently and to answer a true/false comprehension question that probed the interpretation of the sentence they had just read. Comprehension questions occurred after every sentence and were answered by pressing “x” for true and “m” for false on a Standard English keyboard. The study was self-paced such that participants could take a break as needed (between trials) and were recalibrated if a break was taken. Recalibration also took place as needed, and obligatorily halfway through the study.

### Analysis

Prior to analysis, 14.41% of the trials were eliminated for one of two reasons. First, a saccade was made across the boundary but the fixation landed on a pre-target word (i.e., j-hook). Second, a fixation was made on the target word before the boundary change occurred. Comprehension accuracy was relatively high (L1: 92%, L2: 89%), and will not be considered further.

Two duration-based eye movement measures were analyzed: first fixation duration (FFD) and gaze duration (GD). FFD is the duration of the first fixation within the region of interest. GD is the duration of fixations on the region of interest before the eye moves to another word. If the region of interest is skipped during first pass reading, both FFD and GD are scored as missing value. Given the increased potential for statistical error with multiple comparisons we set an alpha threshold of 0.01 (see also 29).

Data were trimmed prior to inferential analysis: FFD and GD under 80 ms or over 1000 ms were removed (L1: 5.76% for FFD and 5.94% for GD, L2: 5.87% for FFD and 6.55% for GD). All dependent variables were analyzed with generalized linear mixed-effects models (GLMM) using the lme4 package (30) in R (31), and results include p-value estimates from the lmerTest package (32). Given that duration measures are always positively skewed, the models for the duration measures were specified with an identity link function (this specifies a linear relationship between predictors and observed responses) and a Gamma distribution (this specifies that the durations are all positive) (see 33).

The fixed effects consisted of two scaled (to reduce collinearity) continuous predictors (spelling score and OPT score) as main effects, mask type (Identical/EPW/GPW/Illegal/X/Blank), language (English/German) and the interaction between mask type and language. Two models were fit for each measure. The first used successive difference contrasts in which each level of masking was compared to the following level (Identical vs. EPW, EPW vs. GPW, GPW vs. Illegal, Illegal vs. X, X vs. Blank). The second used treatment contrast coding with the Identical level set as the reference level to which the five uninformative masks were compared. In both models deviation contrast coding was used for language (.5/-.5). The random effects structure was maximally specified (34) with the random intercepts by participant including random slopes of mask type, and random intercepts by item including random slope for both language and mask type (due to convergence errors, the interaction between mask type and language was removed.). Omnibus tests for the main effects of mask type and language and their interaction were run using log likelihood ratio tests comparing the full model to a model excluding the effect of interest. Below, t and p values are reported; see Appendix 2 for additional model information, and see Table 3 for mean values and standard deviations. The code and data are available at https://osf.io/396a4/.

Skipping rate was the percentage of trials where the region of interest was skipped during first pass reading. Given that skipping rate is binary (whether the word is skipped or not), analyses of this variable were based on binary logistic GLMM. However, an additional covariate (scaled to reduce collinearity) was added, namely the character length of the mask. Below, z and p values are reported; see Appendix 2 for additional model information, and see Table 3 for mean values and standard deviations. The code and data are available at https://osf.io/396a4/.

**Table 3.** Means and Standard Deviations per Measure and Condition

L1 English						
	FFD (ms)		GD (ms)		Skipping (%)	
Condition	M	SD	M	SD	M	SD
Identical	215	76	245	122	11	0.32
EPW	221	92	258	132	8	0.27
GPW	230	83	267	119	5	0.23
Illegal	240	105	277	133	6	0.25
X	277	125	329	157	7	0.25
Blank	223	97	298	174	17	0.38

**Table 3 to be continued:** 

L2 English						
	FFD (ms)		GD (ms)		Skipping (%)	
Condition	M	SD	M	SD	M	SD
Identical	227	80	278	129	10	0.31
EPW	244	95	320	142	10	0.30
GPW	239	98	306	138	10	0.31
Illegal	249	104	310	169	11	0.32
X	277	109	351	171	15	0.36
Blank	246	77	319	147	13	0.34

Note. (FFD = First Fixation Duration; GD = Gaze Duration; Skipping = Skipping Rate)..

## Results

### First fixation duration

**Figure 1.**
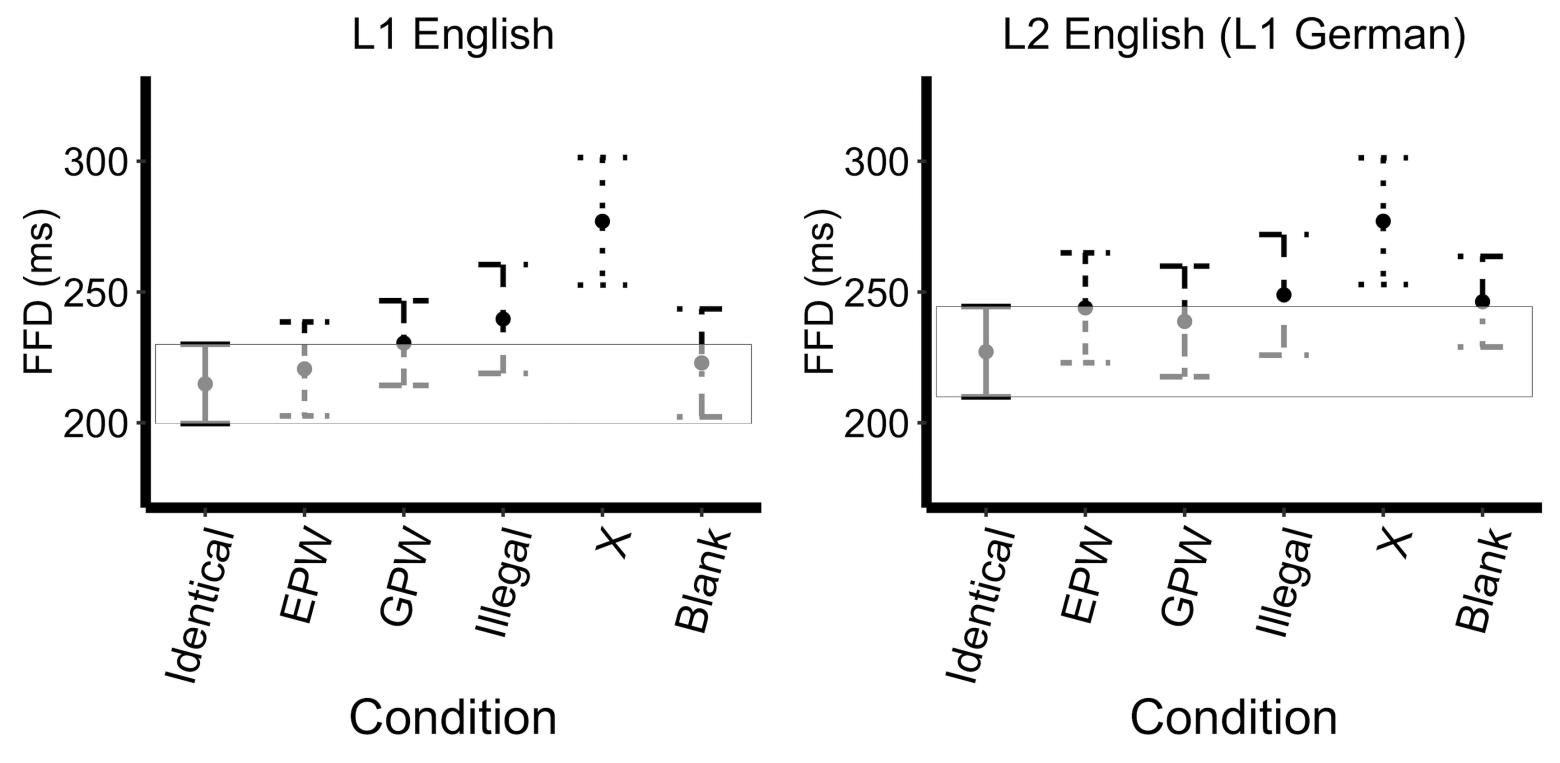
First Fixation Duration Across Uninformative Masks. Error bars represent 99% confidence intervals (the box encompasses the confidence interval of the Identical mask reference condition).

Likelihood-ratio model comparison revealed a main effect of mask type approaching significance (*X^2^*(5)=9.78, *p=*0.08), a main effect of language (*X^2^*(1)=24.67, *p<*0.0001) and a no interaction (*X^2^*(5)=0.59, *p=*0.99). As shown by the successive comparisons, the FFD increased from the Illegal mask to X mask (*t*=3.01, *p*<0.01), with FFD and decreased significantly from the X mask to the Blank mask (*t*=-3.70, *p*<0.001). The model with treatment contrasts (Identical set as the reference level) revealed that both the Illegal mask (*t*=3.05, *p*<0.01) and the X mask (*t*=6.01, *p*<0.0001) evoked a significantly greater FFD than the Identical mask, see Figure 1. No further effects reached significance; see Appendix 2.

### Gaze Duration

**Figure 2.**
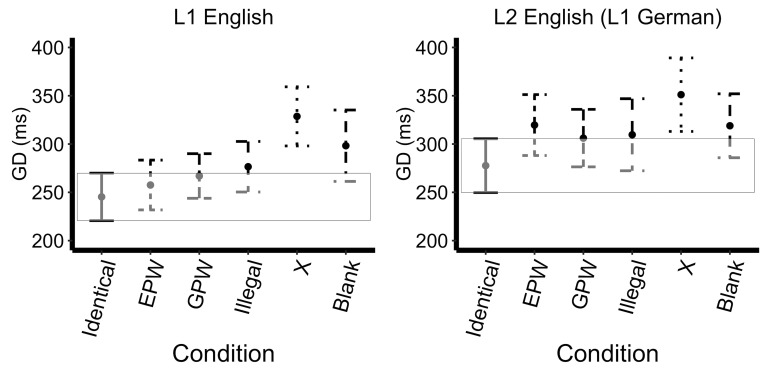
Gaze Duration Across Uninformative Masks. Error bars represent 99% confidence intervals (the box encompasses the confidence interval of the Identical mask reference condition).

Likelihood-ratio model comparisons revealed a main effect of mask type (*X^2^*(5)=64.46, *p<*0.0001), a main effect of Language (*X^2^*(1)=49.09, *p<*0.0001) but no significant interaction (*X^2^*(5)=7.44, *p=*0.19). As shown by the successive comparisons, the GD increased from the Identical to the EPW mask (approaching significance *t*=2.15, *p*=0.03), the Illegal to the X masks *t*=3.55, *p*<0.001), and decreased from the X to the Blank mask (approaching significance, *t*=-2.13, *p*=0.03), with GD decreasing. Additionally, L2 speakers showing greater average GDs than L1 speakers (*t*=2.73, *p*<0.01). The model with the treatment contrasts (Identical set as the reference level) revealed that the Identical mask had a significantly shorter GD relative to all of the other masks: EPW (*t*=2.34, *p*=0.02, approaching significance), GPW (*t*=2.59, *p*<0.01), Illegal (*t*=2.47, *p*=0.01), X (*t*=6.02, *p*<0.0001), Blank (*t*=2.84, *p*<0.01). The L2 speakers showed a greater average GDs than L1 speakers (*t*=-2.36, *p*=0.02, approaching significance); see Figure 2. No further effects reached significance in GD; see Appendix 2.

### Skipping rate

For skipping rate, the maximal model failed to converge and the slope for mask type was removed from the by-item random effects. Model comparison revealed a main effect of mask type (*X^2^*(5)=17.96, *p<*0.01), but no significant effect of language (*X^2^*(1)=0.00, *p=*1.00) or interaction (*X^2^*(5)=9.18, *p=*0.10). As shown by the successive comparisons, skipping rate decreasing from the Identical to the EPW mask (*z*=-2.71, *p*<0.01), increasing from the GPW to Illegal (*z*=2.27, *p*=0.02, approaching significance), and increasing from the Illegal to the X mask (*z*=2.60, *p*<0.01). Additionally, all covariates approached significance: skipping rate decreased as character length increased (*z*=-2.27, *p*=0.02), skipping rate decreased as OPT score increased (*z*=-1.94, *p*=0.05), and skipping rate increased as spelling score increased (*z*=1.94, *p*<0.05).

**Figure 3.**
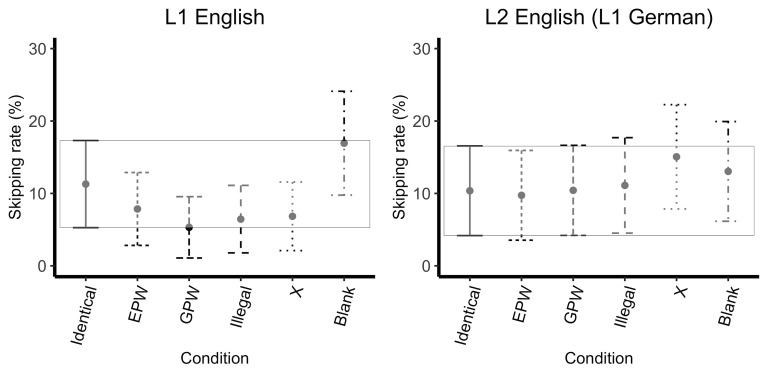
Skipping Rate (%) Across Uninformative Masks. Error bars represent 99 % confidence intervals (the box encompasses the confidence interval of the Identical mask reference condition)

The model with the treatment contrasts (Identical factor set as the reference level) revealed that the Identical mask was skipped more often relative to the EPW (*z*=-2.71, *p*<0.01), GPW (*z*=-4.09, *p*<0.0001), and Illegal masks (*z*=-2.01, *p*=0.04, approaching significance), and was skipped less often relative to the Blank mask (*z* =2.25, *p*=0.02, approaching significance), which was skipped more than the Identical mask; see Figure 3. Additionally, all covariates approached significance: skipping rate decreased as character length increased (*z*=-2.27, *p*=0.05), skipping rate decreased as OPT score increased (*z*=-1.94, *p*=0.05), and skipping rate increased as spelling score increased (*z*=1.94, *p*<0.05). Nothing else reached significance; see Appendix 2.

## Discussion

In this reading study, we investigated the role of uninformative masks on the N+1 preview effect by both L1 and L2 speakers of English. We had three objectives. The first was to test the role of uninformative parafoveal masks in N+1 processing. The second was to test whether L2 speakers of English (with German L1) are similarly influenced by uninformative parafoveal masks relative to L1 English speakers. The third objective was to test whether L1 and L2 speakers were sensitive to parafoveally viewed language-specific sub-lexical orthographic information. We used various types of uninformative masks as a tool to achieve these objectives: (a) an Identical mask, (b) an English pseudo-word, (c) a German pseudo-word, (d) an Illegal string of letters, (e) a series of X’s, and (f) a Blank mask.

If all uninformative masks were equally effective, we would expect the same amount of N+1 interference regardless of mask type. However, it is clear from the findings in the current study that different types of uninformative masks affect reading durations differently. We will begin with discussing how uninformative masks impact on N+1 processing, and then discuss L1/L2 N+1 effects. This is followed by a section on the role of language-specific sub-lexical orthographic information. We then briefly discuss individual differences before our concluding remarks.

### Role of Uninformative Masks on N+1 Processing

In their meta-analysis Vasilev & Angele (2017) argue that N+1 interference effects are smallest for unrelated words, followed by pseudo-words, then random strings of letters, and finally string of X’s (6). Based on this, we hypothesized a rank order of interference (from least to most) of Pseudo-words (potentially language specific) < Illegal < X’s ≤ Blank. We found that the pseudo-word, Illegal, and Blank masks showed relatively similar interference, while the X masks evoked the greatest N+1 interference. When looking at first fixation duration (FFD) and gaze duration (GD), similar patterns emerge, but there are also some important differences. In terms of similarities, both FFD and GD show an increase in duration from the Illegal mask to the X mask, and a decrease in duration from the X mask to the Blank mask (approaching significance for gaze duration). This suggests that parafoveal masks consisting of only X’s cause the greatest disruption when the critical word is ultimately fixated on (relative to the other uninformative masks). For the case where there is pure whitespace in the parafoveal area (Blank mask condition) we tentatively hypothesized that this would lead to greater interference. However, it became clear from our data that the Blank mask condition causes less interference than a mask consisting of a series of X’s. Visual inspection of Figures 1 and 2 and Table 3 suggests that the Blank mask condition is actually not that different to the other non-X mask types, although we did not test this directly.

In terms of the differences between FFD and GD, the EPW evoked a greater N+1 effect in GD (but not in FFD). That is, FFD did not differ between Identical, EPW, GPW, and Illegal masks, but increased from Illegal to the X mask (Pattern: Identical = EPW = GPW = Illegal < X). This suggests that the more “word-like” of the masks (Identical, Pseudo-word, and Illegal) were not distinguished much in FFD, while the X mask led to a clear increase in interference. GD, on the other hand, increased from Identical to EPW masks (marginally significant, *p*=0.03), but did not differ between EPW, GPW, and Illegal masks, and increased from Illegal to X masks (Pattern: Identical < EPW = GPW = Illegal < X). In other words, GD increased as soon as a non-Identical mask was used, then did not differ among the Pseudo-word or Illegal masks, but increased again for the X masks. This suggests that when fixating on a critical word that has been parafoveally denied by an uninformative mask, more “word-like” masks (EPW, GPW, Illegal) do not cause immediate disruption on the first fixation, but do lead to an increase in additional fixations on the critical word before moving on. The X mask, on the other hand, leads to both an immediate disruption in FFD and an additional increase in GD before moving on.

This pattern is also partially confirmed in the treatment contrast models for FFD and GD. The FFD on the critical word following an Identical mask was significantly shorter than following an Illegal mask or following an X mask, while the GD after an Identical mask was shorter than every other mask type. This suggests that first fixations on the critical word were most disrupted by less word-like masks, but additional fixations on the critical word were impacted by all types of uninformative masks.

Skipping rate decreased from Identical to EPW, and increased both from GPW to Illegal (marginally significant, *p*=0.02) and from X to Blank masks. The additional predictor of character length was also marginally significant (*p*=0.02), with skipping decreasing as character length increased. Skipping rate in the Identical mask condition was significantly greater than for all other mask types except the Blank mask condition, where skipping rate was even higher than in the Identical mask condition. This suggests that speakers were less likely to skip words that were masked by Pseudo-words and slightly more likely to skip words that were masked by less word-like strings of letters. When a parafoveal mask is entirely absent, participants will skip the Blank area entirely. Intuitively this makes sense, since when there is a blank space in the parafoveal area, the reader may program a saccade to the next available word while fixating on the foveal word.

When comparing the observed pattern of interference with the rank order put forward by Vasilev and Angele (unrelated word < pseudo words < strings of letters < string of X’s) and our hypothesis (Pseudo-words (potentially language specific) < Illegal < X’s ≤ Blank), the statistical evidence obtained in the present study leads us to revise the continuum to something like: Identical < EPW = GPW = Illegal = Blank < X’s (6). What is quite robust is that the X mask evokes the greatest interference, with slightly graded differences between the other masks. This is in line with previous research that suggests parafoveal masks consisting of strings of X’s lead to a N+1 preview interference effect (e.g., 7, 8). As Vasilev and Angele point out, the interference from uninformative masks may only amount to a few millisecond in first-pass measures, and we see interference effects anywhere from 6ms to 83ms (with the least inference typically coming form the Pseudo-word masks and the most interference always coming from the X masks) (6). The lack of significance in the continuum may stem from these potentially small effects and may be more robust with more items per condition (see the limitations section).

When calculating the N+1 benefit, researchers traditionally compare the reading time of a critical word after it has been parafoveally masked with some meaningful mask (i.e., orthographic, phonological, morphological, or semantic) relative to when the same critical word has been parafoveal masked with some uninformative mask (e.g., unrelated word, psuedo-word, string of letters, or a series of X’s). To date, the types of masks that a researcher chooses to compare to calculate a N+1 effects seem to have little theoretical grounding, and this may be inflating or obscuring true N+1 effects. It is clear from the current study that research using uninformative parafoveal masks should be careful to choose a mask that will not inflate the N+1 preview effect, and should be aware that, in particular, X masks lead to the largest N+1 interference.

### N+1 Effects in L1 versus L2

Using language to communicate is a fundamental part of being human, and literacy is a key component in functioning societies given the sheer amount of information that is conveyed in written form. However most of our understanding of reading and language processing is based on monolingual speakers of English, despite the reality that more than half of the world speaks more than one language (35). To our knowledge, there are only three studies that investigate parafoveal processing by L2 speakers (20, 21, 22) and two of the three studies focus on semantic parafoveal processing rather than bilingualism per se. Given the evidence that denying parafoveal information to L1 speakers leads to reading disruptions, it is important that we understand the way in which L2 speakers use parafoveal information, in order to better understand L2 sentence processing. The current research not only adds to the limited research investigating L2 parafoveal processing, and but also adds to the L1 literature by systematically manipulating uninformative mask types.

We found that the L1 and L2 groups behaved quite similarly in response to the experimental manipulations in our study. There was only one clear difference between the groups: GDs were slightly shorter in L1 readers than L2 readers. While there were some more subtle differences between the two groups, the data overall suggest that L1 and L2 speakers are similarly affected by uninformative masks. This study extends the current literature and suggests that even in languages with an opaque spelling to sound correspondence (like English), L2 speakers are able to engage in the early preattentional “visual check” stage (12, 13). Whether L2 speakers are engaging in the attention-dependent lexical processing stage is not clear, and should be investigated further.

Another important difference between L1 and L2 speakers was found for skipping rate, as seen in Figure 3. In particular, the L2 group, for almost all conditions, skipped words more than the L1 group, and at a relatively higher rate. While L2 eye-movement research has reported skipping rate (in conjunction with other measures), no research has specifically investigated L2 skipping rate during reading, to the knowledge of the authors. Research that has reported L2 skipping rate seems to suggest that L2 speakers have a lower skipping rate than L1 speakers (e.g., 36). The three studies noted earlier that investigated L2 parafoveal processing found lower skipping rates than those reported here, and also found that readers showed some sensitivity to different mask types. Only 0.05% of the target words were skipped (with no difference by mask type) in Wang et al. (2014), and 3% of target words were skipped in Wang et al. (2016) with participants skipping target words that had a cognate parafoveal mask more than the other mask types (22, 21). Altarriba et al. reported a higher skipping rate, with identical preview evoking the largest skipping rate (8.3%) and participants skipping the other mask types less frequently (20). One potential explanation for the difference between the present study and the others that found lower skipping rates is that the parafoveal word was expected in our study, while the other studies had neutral sentence contexts so the parafoveal word was not expected. This suggests that L2 speakers consistently skip expected words regardless of mask type. It is possible that L2 speakers are employing a “riskier” reading strategy similar to what has been seen with older readers (>70 years of age). Rayner, Reichle, Stroud, Williams, and Pollatsek (2006) found that older readers were more likely to skip words and relied on only partial information to build expectations of upcoming information. It may be that in the face of a constraining context, L2 speakers are more likely to skip an expected word regardless of whether the parafoveal information was uninformative (37). This should be tested further.

Parafoveal processing and skipping behavior has played an important role in forming L1 models of eye-movement behavior during reading (e.g., SWIFT (38) and the E-Z reader (39), but seems to play little role in models of L2 reading or processing. To the knowledge of the authors, only one study has discussed L2 eye-movements during reading within the context of these models. Cop, Drieghe, & Duyck (2015) investigated eye movements during reading by late bilingual L1 Dutch/L2 English and monolingual L1 English speakers while reading a novel (in English for monolinguals and half in English and half in Dutch for bilinguals) (36). Their data, particularly the decreased skipping rate when reading in an L2 relative to an L1, lent itself to the E-Z reader model. They argued that L2 speakers take longer to access lexical information and have less resources to devote to parafoveal processing, and are less likely to skip. However, this is not compatible with the current data that shows no differences in skipping behavior; we hesitate to make any claims of support of one model over the other based on skipping rate without more systematic investigation.

Further, models of L1 eye-movement behavior during reading do not take L2 eye movement behavior into account. Models of eye-movements during reading should not only explain L1 patterns of behavior, but also L2 patterns of behavior as well, since L2 patterns of behavior may shed important light on L1 behavior. We hope that the current research will give impetus to rectifying this discrepancy and researchers will start approaching language processing and reading behavior not only from a monolingual but also from a multilingual perspective.

### Language-specific Sub-lexical Orthographic Information

The two pseudo-word types allowed us to test whether N+1 interference was language-specific, or more specifically, whether there is less interference from pseudo-words that are more similar to the native language of the participant. As discussed above, there were no reliable graded differences between the two types of pseudo-words in any of the measures or in either analysis. This suggests that neither L1 nor L2 speakers are sensitive to parafoveally viewed language-specific sub-lexical orthographic information. This finding contrasts with that of Lemhöfer and Radach (2009), who found that German dominant German-English bilinguals were quicker to reject non-words that were more “German-like” than non-words that were more “English-like” in a lexical decision task (27). Our results may put this finding into perspective, by suggesting that sensitivity to the orthographic regularities of the given language does not arise during the earliest “visual check” stage of (parafoveal) processing, but is likely to emerge during later stages of lexical decision which involve deeper orthographic analysis.

While L1 and L2 speakers did not show statistically significant differences in sensitivity to language-specific sub-lexical properties in the parafovea, Figures 1, 2, and 3 reveal a pattern that suggests a slight sensitivity. Specifically, the L1 speakers have a larger FFD and GD, and a smaller skipping rate following the GPW mask relative to the EPW, while the L2 speakers showing the opposite pattern with a slightly larger FFD and GD and a smaller skipping rate following the EPW relative to the GWP. Given the low number of items per condition in the current study (see the limitations section below) we hesitate to make any claims on the basis of null results, and encourage further investigation of parafoveal sub-lexical orthographic information with more observations per condition. Given that both English and German are Germanic languages it would be interesting test whether pseudo-words from different language families would show more pronounced interference patterns.

### Individual Differences

In terms of individual differences, we controlled for proficiency and spelling skills. Veldre and Andrews (2014) found that L1 English speakers with better reading and spelling skills were more efficient at using parafoveal information, and also experienced greater disruption in reading measures when the parafoveal area was restricted, relative to participants with lower reading and spelling skills (25). Whitford & Titone (2015) also found that L2 speakers with a higher quality of lexical representation (as measured by exposure rate) were more efficient at extracting parafoveal information (19). Further, Wang et al. (2014) found that L2 speakers with higher proficiency were more efficient at extracting parafoveal information (22). Given that these individual differences play a role in the efficiency with which L1 and L2 speakers extract parafoveal information we included them as control predictors in our analyses.

Our results showed that neither spelling skills nor proficiency had a significant effect for the duration measures. However, both approached significance in affecting the skipping rate: skipping rate increased as spelling score increased, and skipping rate decreased as proficiency (OPT score) increased. This suggests that better spellers are more likely to skip words, while more proficient individuals are less likely to skip words. A tentative hypothesis is that better spellers are more efficient at extracting parafoveal information, and thus have less need to actually fixate on the critical word once they have the opportunity to do so. Less proficient individuals, on the other hand, may overly rely on contextual information (in line with the “risker” reading strategy mentioned previously), and thus are more likely to skip words. Given that both spelling and proficiency scores neared significance, and that we did not assess whether these scores interacted with the other fixed factors, it is hard to tell the locus of these differences (for example, spelling differences could be driven by the identical condition, while proficiency differences could be driven by the masked condition, or vice versa). What our results do highlight, however, is the importance of controlling for these factors and investigating them in future research.

### Limitations

We believe this study has one main limitation, which is that the power of the study may be viewed as less than ideal. While the participant sample was reasonably large, the number of items per condition was relatively low. We encourage future research with more items per condition. This may be particularly relevant for the sub-lexical orthographic differences, which showed the hypothesized pattern but did not reach significance. Greater power might lead to more robust differences across the different mask types, and may show clearer individual difference patterns. Despite the potential limitation arising from the items per condition, we believe that this paper has important implications in terms of designing uninformative masks for GCB studies, and also highlights the need for more in-depth and systematic research of L2 reading research, which is surprisingly limited given the prevalence of bi-/multilingual readers.

### Conclusions

Although it is intuitive that less “word-like” uninformative parafoveal masks will interfere with reading more than more “word-like” uninformative parafoveal masks, this has never been systematically tested. Therefore, we tested this hypothesis across both L1 and L2 speakers of English using the gaze contingent boundary paradigm. Two central findings emerged. First, X masks interfered the most with typical reading patterns, with graded differences the less word-like a word becomes (i.e., illegal words, pseudo-words). Second, L1 and L2 speakers were similarly impacted by the various types of uninformative masks. The sub-lexical information within the pseudo-word masks seemed to play little role, and suggests that L1 and L2 speakers are not sensitive to parafoveally-viewed language-specific sub-lexical orthographic information in the early preattentional stages of parafoveal processing.

Our results also have two important implications for the field of parafoveal processing in general. First, in designing future GCB parafoveal processing studies, it is important for researchers to choose parafoveal masks that will not inflate or obscure the effect of the parafoveal manipulation in question. Researchers should be equally as thoughtful and theory-driven about designing uninformative masks as they are about designing informative masks. Second, L1 reading models should take L2 reading behavior into account, and L2 reading research should take parafoveal processing into account. This not only has large practical implications in terms of the educational process of learning a L2, learning in an L2, and communicating in a L2, but also theoretical implications in terms of models of reading behavior and models of sentence processing. We believe that it is important that future research start approaching language not only from a monolingual but also a multilingual perspective.

### Ethics and Conflict of Interest

The author(s) declare(s) that the contents of the article are in agreement with the ethics described in http://biblio.unibe.ch/portale/elibrary/BOP/jemr/ethics.html and that there is no conflict of interest regarding the publication of this paper.

## Appendix

### Appendix 1 – Critical stimuli

The masks are [Identical, English pseudo-word, German pseudo-word, Illegal, X, and blank]

**Table:**

1	The surgeon described the [bone, gice, gein, mkrj, xxxx, ] to his colleagues.	
2	The curator hung the [image, stoze, spoch, cbrkq, xxxxx, ] with great care.	
3	The biologist dissected the [heart, stoth, susid, jqcml, xxxxx, ] in the lab.	
4	The landscaper tidied the [yard, gath, gane, lgkv, xxxx, ] on the estate.	
5	The lawyer consulted the [book, kide, kast, zxgh, xxxx, ] to help with the case.	
6	The lifeguard monitored the [beach, mized, meife, pkrlw, xxxxx, ] and the ocean.	
7	The professor lost the [test, fank, folz, nplg, xxxx, ] before class.	
8	The athlete passed the [ball, lape, laum, pltw, xxxx, ] at school yesterday.	
9	The baker made the [cake, homp, henk, kdtr, xxxx, ] for the party.	
10	The electrician fixed the [light, rudic, rolpe, cjprt, xxxxx, ] for his neighbor.	
11	The musician wrote the [songs, bacel, bauns, fkqxj, xxxxx, ] on the computer.	
12	The housekeeper washed the [dress, bicer, breif, hgjpv, xxxxx, ] for the woman.	
13	The pirate brought the [chest, pault, pahme, nglkj, xxxxx, ] aboard the old leaky ship.	
14	The family lived in the [house, snode, stebs, fpktx, xxxxx, ] across town.	
15	The painter added the [white, urage, ulsel, fmlrp, xxxxx, ] to the portrait.	
16	The dentist inspected the [mouth, snosh, stort, kwgfp, xxxxx, ] with care.	
17	The chef made the [dinner, geason, gaflik, fzxtlh, xxxxxx, ] to surprise his friends.	
18	The botanist studied the [plants, snirge, spreme, wqlvrf, xxxxxx, ] to learn more.	
19	The researchers consulted the [experts, bealing, blossig, nvbwzx, xxxxxxx, ] about the project.	
20	The patient found the [doctor, shiple, sittam, gplskv, xxxxxx, ] in the parking lot.	
21	The waiter brought the [dishes, strile, stralt, jlxwtq, xxxxxx, ] to the customer.	
22	The gardener picked the [flower, nuncer, nekien, gqzlsj, xxxxxx, ] to give to his partner.	
23	The patient found the [doctor, shiple, sittam, gplskv, xxxxxx, ] in the parking lot.	
24	The geologist found the [rock, mish, mand, nbpl, xxxx, ] in the cave.	

### Appendix 2 – Mixed Model Information

First Fixation Model Parameters

**Table:**

Successive comparisons	Estimate	Std. Error	t value	Pr(>|t|)
**Intercept**	**232.71**	**3.62**	**64.30**	**<0.0001**
English pseudo-word vs Identical	8.95	8.60	1.04	0.30
German pseudo-word-vs English pseudo-word	0.83	8.79	0.10	0.92
Illegal vs German pseudo-word	9.46	9.04	1.05	0.30
**X vs Illegal**	**31.10**	**10.32**	**3.01**	**<0.01**
**Blank vs X**	**-37.59**	**10.15**	**-3.70**	**<0.001**
English	-11.36	9.58	-1.19	0.24
Spelling	0.10	0.44	0.22	0.83
OPT	-0.24	0.97	-0.25	0.80
English pseudo-word vs Identical: English	-9.00	15.91	-0.57	0.57
German pseudo-word-vs English pseudo-word: English	20.02	16.18	1.24	0.22
Illegal vs German pseudo-word: English	-7.49	16.21	-0.46	0.64
X vs Illegal: English	11.10	19.34	0.57	0.57
Blank vs X: English	-27.88	17.79	-1.57	0.12
				
Identical reference level	Estimate	Std. Error	t value	Pr(>|t|)
**Intercept (Identical)**	**222.34**	**6.41**	**34.70**	**<0.0001**
English pseudo-word	12.25	9.22	1.33	0.18
German pseudo-word	12.03	8.05	1.49	0.14
**Illegal**	**26.48**	**8.68**	**3.05**	**<0.01**
**X**	**61.82**	**10.28**	**6.01**	**<0.0001**
Blank	14.25	8.59	1.66	0.10
English	-10.55	12.97	-0.81	0.42
Spelling	0.14	0.44	0.32	0.75
OPT	-0.29	0.96	-0.30	0.77
English pseudo-word: English	-8.18	16.39	-0.50	0.62
German pseudo-word: English	9.30	14.62	0.64	0.52
Illegal: English	6.31	15.16	0.42	0.68
X: English	16.32	18.18	0.90	0.37
Blank: English	-14.79	15.83	-0.94	0.35

**Table:**

Gaze duration Model Parameters				
Successive comparisons	Estimate	Std. Error	t value	Pr(>|t|)
**Intercept**	**283.15**	**5.85**	**48.36**	**<0.0001**
**English pseudo-word vs Identical**	**26.35**	**12.27**	**2.15**	**0.03***
German pseudo-word-vs English pseudo-word	-1.39	10.46	-0.13	0.89
Illegal vs German pseudo-word	3.76	10.89	0.35	0.73
**X vs Illegal**	**46.24**	**13.03**	**3.55**	**<0.001**
**Blank vs X**	**-35.70**	**16.76**	**-2.13**	**0.03***
**English**	**-41.11**	**15.03**	**-2.73**	**<0.01**
Spelling	-0.95	0.68	-1.40	0.16
OPT	1.87	1.49	1.25	0.21
English pseudo-word vs Identical: English	-25.38	21.31	-1.19	0.23
German pseudo-word-vs English pseudo-word: English	24.97	19.88	1.26	0.21
Illegal vs German pseudo-word: English	7.64	20.18	0.38	0.71
X vs Illegal: English	8.10	25.90	0.31	0.75
Blank vs X: English	-8.46	27.68	-0.31	0.76
Identical reference level	Estimate	Std. Error	t value	Pr(>|t|)
**Intercept (Identical)**	**253.79**	**7.70**	**32.96**	**<0.0001**
**English pseudo-word**	**25.00**	**10.68**	**2.34**	**0.02***
**German pseudo-word**	**22.68**	**8.77**	**2.59**	**<0.01**
**Illegal**	**25.13**	**10.17**	**2.47**	**0.01**
**X**	**72.91**	**12.12**	**6.02**	**<0.0001**
**Blank**	**37.66**	**13.28**	**2.84**	**<0.01**
**English**	**-42.25**	**17.91**	**-2.36**	**0.02***
Spelling	-0.71	0.68	-1.04	0.30
OPT	1.34	1.50	0.89	0.37
English pseudo-word: English	-24.13	20.33	-1.19	0.24
German pseudo-word: English	1.97	17.53	0.11	0.91
Illegal: English	8.56	18.99	0.45	0.65
X: English	16.80	23.11	0.73	0.47
Blank: English	9.10	22.49	0.41	0.69

Skipping Rate Model Parameters

**Table:**

Successive comparisons	Estimate	Std. Error	z value	Pr(>|t|)
**Intercept**	**-3.04**	**0.18**	**-17.29**	**<0.0001**
**English pseudo-word vs Identical**	**-0.87**	**0.64**	**-2.71**	**<0.01**
German pseudo-word-vs English pseudo-word	-0.68	0.85	-1.62	0.11
**Illegal vs German pseudo-word**	**0.95**	**0.84**	**2.27**	**0.02***
X vs Illegal	0.48	0.59	1.63	0.10
**Blank vs X**	**0.71**	**0.55**	**2.60**	**<0.01**
English	0.00	0.46	0.00	1.00
**Spelling**	**0.04**	**0.02**	**1.94**	**0.05***
**OPT**	**-0.08**	**0.04**	**-1.94**	**0.05***
**Mask length**	**-0.29**	**0.13**	**-2.27**	**0.02***
English pseudo-word vs Identical: English	-0.37	1.28	-0.58	0.56
German pseudo-word-vs English pseudo-word: English	-0.82	1.69	-0.97	0.33
Illegal vs German pseudo-word: English	0.27	1.68	0.32	0.75
X vs Illegal: English	-0.31	1.19	-0.52	0.60
**Blank vs X: English**	**1.34**	**1.10**	**2.45**	**0.014***
				
Identical reference level	Estimate	Std. Error	z value	Pr(>|t|)
**Intercept (Identical)**	**-2.62**	**0.22**	**-11.82**	**<0.0001**
**English pseudo-word**	**-0.87**	**0.64**	**-2.71**	**<0.01**
**German pseudo-word**	**-1.55**	**0.76**	**-4.09**	**<0.0001**
**Illegal**	**-0.60**	**0.60**	**-2.01**	**0.04***
X	-0.12	0.53	-0.44	0.66
**Blank**	**0.60**	**0.53**	**2.25**	**0.02***
English	0.60	0.53	1.13	0.26
**Spelling**	**0.04**	**0.02**	**1.94**	**0.05***
**OPT**	**-0.08**	**0.04**	**-1.94**	**0.05***
**Mask length**	**-0.29**	**0.13**	**-2.27**	**0.02***
English pseudo-word: English	-0.37	1.28	-0.58	0.56
German pseudo-word: English	-1.19	1.52	-1.56	0.12
Illegal: English	-0.92	1.19	-1.54	0.12
**X: English**	**-1.23**	**1.07**	**-2.30**	**0.02***
Blank: English	0.12	1.06	0.22	0.83

## References

[b20] Altarriba, J., Kambe, G., Pollatsek, A., & Rayner, K. (2001). Semantic codes are not used in integrating information across eye fixations in reading: Evidence from fluent Spanish-English bilinguals. Perception & Psychophysics, 63, 875–890. 10.3758/BF03194444 0031-5117 11521853

[b12] Angele, B., Tran, R., & Rayner, K. (2013). Parafoveal-foveal overlap can facilitate ongoing word identification during reading: Evidence from eye movements. Journal of Experimental Psychology. Human Perception and Performance, 39, 526–538. 10.1037/a0029492 0096-1523 22866764 PMC3596446

[b13] Angele, B., Slattery, T. J., & Rayner, K. (2016). Two stages of parafoveal processing during reading: Evidence from a display change detection task. Psychonomic Bulletin & Review, 23, 1241–1249. 10.3758/s13423-015-0995-0 1069-9384 26769246 PMC4974265

[b5] Balota, D. A., Pollatsek, A., & Rayner, K. (1985). The interaction of contextual constraints and parafoveal visual information in reading. Cognitive Psychology, 17, 364–390. 10.1016/0010-0285(85)90013-1 0010-0285 4053565

[b34] Barr, D. J., Levy, R., Scheepers, C., & Tily, H. J. (2013). Random effects structure for confirmatory hypothesis testing: Keep it maximal. Journal of Memory and Language, 68, 255–278. 10.1016/j.jml.2012.11.001 0749-596X 24403724 PMC3881361

[b30] Bates, D. M., Maechler, M., & Bolker, B. lme4: Linear mixed-effects models using S4 classes. R package ver-sion 1.1-18-1 [software]. 2018. Available from:https://cran.r-project.org/web/packages/lme4/index.html

[b36] Cop, U., Drieghe, D., & Duyck, W. (2015). Eye movement pat-terns in natural reading: A comparison of monolingual and bilingual reading of a novel. PLoS One, 10, e0134008. 10.1371/journal.pone.0134008 1932-6203 26287379 PMC4545791

[b38] Engbert, R., Nuthmann, A., Richter, E. M., & Kliegl, R. (2005). SWIFT: A dynamical model of saccade generation during reading. Psychological Review, 112, 777–813. 10.1037/0033-295X.112.4.777 0033-295X 16262468

[b15] Fernandez LB, Bothe ER, Allen SEM. The role of L1 reading direction on L2 perceptual span: An eye tracking study investigating Hindi and Urdu speakers. Under review.

[b7] Hutzler, F., Fuchs, I., Gagl, B., Schuster, S., Richlan, F., Braun, M., & Hawelka, S. (2013). Parafoveal X-masks interfere with foveal word recognition: Evidence from fixation-related brain potentials. Frontiers in Systems Neuroscience, 7, 1-10. 10.3389/fnsys.2013.00033 1662-5137 23888130 PMC3719217

[b9] Hutzler, F., Schuster, S., Marx, C., & Hawelka, S. (2018). An inves-tigation of parafoveal masks with the incremental boun-dary paradigm. PLoS One, 14, 1–26.1932-6203 10.1371/journal.pone.0203013 PMC6394947 30817789

[b14] Jordan, T. R., Almabruk, A. A. A., Gadalla, E. A., McGowan, V. A., White, S. J., Abedipour, L., & Paterson, K. B. (2014). Reading direction and the central perceptual span: Evidence from Arabic and English. Psychonomic Bulletin & Review, 21, 505–511. 10.3758/s13423-013-0510-4 1069-9384 24065283

[b28] Keuleers, E., & Brysbaert, M. (2010). Wuggy: A multilingual pseudoword generator. Behavior Research Methods, 42, 627–633. 10.3758/BRM.42.3.627 1554-351X 20805584

[b10] Kliegl, R., Hohenstein, S., Yan, M., & McDonald, S. A. (2013). How preview space/time translates into preview cost/benefit for fixation durations during reading. Quarterly Journal of Experimental Psychology, 66, 581–600. 10.1080/17470218.2012.658073 1747-0218 22515948

[b32] Kuznetsova, A., Brockhoff, P. B., & Bojesen, C. lmerTest: Tests in linear effects models. R package version 3.0-1 [software]. 2018. Available from:https://cran.r-project.org/web/packages/lmerTest/index.html

[b27] Lemhöfer, K., & Radach, R. (2009). Task context effects in bilingual nonword processing. Experimental Psychology, 56, 41–47. 10.1027/1618-3169.56.1.41 1618-3169 19261577

[b16] Leung, C. Y., Sugiura, M., Daisuke, A., & Yoshikawa, L. (2014). The perceptual span in second language reading: An eye-tracking study using a gaze-contingent moving window paradigm. Open Journal of Modern Linguistics, 4, 585–594. 10.4236/ojml.2014.45051 2164-2818

[b33] Lo, S., & Andrews, S. (2015). To transform or not to transform: Using generalized linear mixed models to analyse reaction time data. Frontiers in Psychology, 6, 1171. 10.3389/fpsyg.2015.01171 1664-1078 26300841 PMC4528092

[b35] Marian V, Shook A. The cognitive benefits of being bilingual. Cerebrum: the Dana forum on brain science 2012;1-11. PMC3583091 23447799

[b8] Marx, C., Hawelka, S., Schuster, S., & Hutzler, F. (2015). An incremental boundary study on parafoveal preprocessing in children reading aloud: Parafoveal masks overestimate the preview benefit. Journal of Cognitive Psychology, 27, 549–561. 10.1080/20445911.2015.1008494 2044-5911 26246890 PMC4487581

[b4] Matin, E. (1974). Saccadic suppression: A review and an analysis. Psychological Bulletin, 81, 899–917. 10.1037/h0037368 0033-2909 4612577

[b17] Paterson, K. B., McGowan, V. A., White, S. J., Malik, S., Abedipour, L., & Jordan, T. R. (2014). Reading direction and the central perceptual span in Urdu and English. PLoS One, 9, e88358. 10.1371/journal.pone.0088358 1932-6203 24586316 PMC3934859

[b24] Perfetti, C. A., & Hart, L. (2001). The lexical bases of comprehen-sion skill. In D. S. Gorfien (Ed.), On the consequences of meaning selection: Perspectives on resolving lexical ambiguity (pp. 67–86). American Psychological Association. 10.1037/10459-004

[b18] Pollatsek, A., Bolozky, S., Well, A. D., & Rayner, K. (1981). Asymmetries in the perceptual span for Israeli readers. Brain and Language, 14, 174–180. 10.1016/0093-934X(81)90073-0 0093-934X 7272722

[b3] Rayner, K. (1975). The perceptual span and peripheral cues in reading. Cognitive Psychology, 7, 65–81. 10.1016/0010-0285(75)90005-5 0010-0285

[b2] Rayner, K. (1998). Eye movements in reading and information processing: 20 years of research. Psychological Bulletin, 124, 372–422. 10.1037/0033-2909.124.3.372 0033-2909 9849112

[b37] Rayner, K., Reichle, E. D., Stroud, M. J., Williams, C. C., & Pollatsek, A. (2006). The effect of word frequency, word predictability, and font difficulty on the eye movements of young and older readers. Psychology and Aging, 21, 448–465. 10.1037/0882-7974.21.3.448 0882-7974 16953709

[b31] R Core Team. R: A language and enviorment for statistical computing. R Foundation for Statistical Com-puting, Vienna, Austria [software]. 2018.Available from: https://www.R-project.org/

[b39] Reichle, E. D., Rayner, K., & Pollatsek, A. (2003). The E-Z reader model of eye-movement control in reading: Comparisons to other models. Behavioral and Brain Sciences, 26, 445–476. 10.1017/S0140525X03000104 0140-525X 15067951

[b1] Schotter, E. R., Angele, B., & Rayner, K. (2012). Parafoveal processing in reading. Attention, Perception & Psychophysics, 74, 5–35. 10.3758/s13414-011-0219-2 1943-3921 22042596

[b26] Schröter, P., & Schroeder, S. (2018). Exploring early language detection in balanced bilingual children: The impact of language-specificity on cross-linguistic nonword recogni-tion. The International Journal of Bilingualism, 22, 305–315. 10.1177/1367006916672751 1367-0069

[b11] Slattery, T. J., Angele, B., & Rayner, K. (2011). Eye movements and display change detection during reading. Journal of Experimental Psychology. Human Perception and Performance, 37, 1924–1938. 10.1037/a0024322 0096-1523 21688934

[b6] Vasilev, M. R., & Angele, B. (2017). Parafoveal preview effects from word N + 1 and word N + 2 during reading: A critical review and Bayesian meta-analysis. Psychonomic Bulletin & Review, 24, 666–689. 10.3758/s13423-016-1147-x 1069-9384 27576520

[b25] Veldre, A., & Andrews, S. (2014). Lexical quality and eye movements: Individual differences in the perceptual span of skilled adult readers. Quarterly Journal of Experimental Psychology, 67, 703–727. 10.1080/17470218.2013.826258 1747-0218 23972214

[b22] Wang, A., Zhou, W., Shu, H., & Yan, M. (2014). Reading proficiency modulates parafoveal processing efficiency: Evidence from reading Chinese as a second language. Acta Psychologica, 152, 29–33. 10.1016/j.actpsy.2014.07.010 0001-6918 25103417

[b21] Wang, A., Yeon, J., Zhou, W., Shu, H., & Yan, M. (2016). Cross-language parafoveal semantic processing: Evidence from Korean-Chinese bilinguals. Psychonomic Bulletin & Review, 23, 285–290. 10.3758/s13423-015-0876-6 1069-9384 26122894

[b19] Whitford, V., & Titone, D. (2015). Second-language experience modulates eye movements during first- and second-language sentence reading: Evidence from a gaze-contingent moving window paradigm. Journal of Experimental Psychology. Learning, Memory, and Cognition, 41, 1118–1129. 10.1037/xlm0000093 0278-7393 25528098

[b23] Yan, M., Zhou, W., Shu, H., & Kliegl, R. (2012). Lexical and sublexical semantic preview benefits in Chinese reading. Exp Psychol Learn Mem Cog, 38, 1069–1075. 10.1037/a0026935 1939-1285 22369254

[b29] von der Malsburg, T., & Angele, B. (2017). False positive rates in standard analyses of eye movements in reading. Journal of Memory and Language, 94, 119–133. 10.1016/j.jml.2016.10.003 0749-596X 28603341 PMC5461930

